# Effect of COVID‐19 Pandemic on Epidemiology and Antimicrobial Susceptibility of *Campylobacter* Species, *Salmonella enterica* and *Yersinia enterocolitica* in Southwest Finland 2018–2022

**DOI:** 10.1111/apm.70187

**Published:** 2026-03-13

**Authors:** Tanja Orpana, Teemu Kallonen, Antti J. Hakanen, Marianne Gunell

**Affiliations:** ^1^ Institute of Biomedicine, University of Turku Turku Finland; ^2^ Turku Clinical Microbiome Bank, Clinical Microbiology, Turku University Hospital, the Wellbeing Services County of Southwest Finland Turku Finland

**Keywords:** antimicrobial resistance, campylobacter, enteropathogens, salmonella, Yersinia

## Abstract

This study investigated the effect of the COVID‐19 pandemic on the epidemiology and antimicrobial susceptibility of fecal *Campylobacter* spp.*, Salmonella enterica*, and *Yersinia enterocolitica* strains in Southwest Finland from 2018 to 2022. Results show that the number of travel‐associated *S. enterica* and *Campylobacter* spp. declined markedly from autumn 2019 to autumn 2020 and have recovered gradually. Antimicrobial susceptibility testing was performed on bacterial strains isolated from PCR‐positive fecal specimens. Resistance patterns fluctuated throughout the study period. Among *C. jejuni* , ciprofloxacin resistance averaged 58% in domestic (*n* = 155) and 88% travel‐associated (*n* = 10) strains, while tetracycline resistance averaged 36% and 63%, respectively; erythromycin resistance was not detected. In *S. enterica* , resistance averaged 42% and 33% to ampicillin, 33% and 45% to fluoroquinolones, 4% and 6% to cefotaxime, and 0% and 2% to co‐trimoxazole, in domestic (*n* = 24) and travel‐associated (*n* = 32) strains, respectively. Among domestic *Y. enterocolitica* strains (*n* = 64), resistance averaged 7% to co‐trimoxazole, 2% to ciprofloxacin, and 1% to cefotaxime; no travel‐associated strains were reported. This study shows that lockdowns due to the COVID‐19 pandemic decreased the number of diagnosed enteropathogens and limited the emergence of resistant strains. Thus, our results reaffirm that travel remains the primary source of *S. enterica* infections in Finland.

## 1 Introduction

Enteropathogenic bacteria, for example *Campylobacter, Salmonella*, and *Yersinia*, are usually zoonotic and spread via contaminated food or water [1, 2]. These bacteria commonly cause mild gastroenteritis, but occasionally severe complications appear, whereupon antimicrobial treatment and even hospitalization are needed [2]. *Campylobacter* spp. are commonly carried in the gut microbiota of healthy broiler chickens [3]. Broiler chicken meat is the most commonly consumed animal protein for human nutrition, and therefore the risks associated with undercooked or raw poultry meat, as well as cross‐contamination from food products, such as campylobacteriosis, are also common [2, 4, 5]. In the European Union, European Food Safety Authority (EFSA) and European Centre for Disease Prevention and Control (ECDC) are responsible for monitoring and surveillance activities for zoonoses such as *Campylobacter* and *Salmonella*. Based on EFSA and ECDC annual surveillance reports, *Campylobacter* is the most reported and *Salmonella* is the second most reported zoonotic bacteria in enteric infections in Europe [6, 7, 8]. In Finland, there is a strict *Salmonella* control policy, which has led to reduced contamination in livestock [1, 9] and in clinical samples over the last decades [10]. The prevalence of *Salmonella* is regularly monitored in livestock in Finland, but only a few studies on the epidemiology of clinical samples have been published in the past 20 years, and most of them focus on reviews of foodborne outbreaks [11, 12, 13, 14, 15, 16, 17, 18, 19]. Clinical *Salmonella* strains discovered in Finland are typically travel‐associated, and fluoroquinolone‐resistant strains are mainly isolated from patients returning from Southeast Asia [17, 19]. *Yersinia enterocolitica* is a cold‐resistant pathogen and grows well in refrigerator temperatures [20]. Infection is typically acquired via raw or undercooked pork meat and can cause yersiniosis, symptoms of which can vary from mild diarrhea to severe invasive infection leading to mesenteric lymphadenitis and abscess formation [20, 21].

Antimicrobial resistance (AMR) in clinical *Campylobacter* sp. and *Salmonella* spp. is monitored at a species level in Finland by the Finnish Study Group for Antimicrobial Resistance (FiRe), a collaborative body consisting of Finnish clinical microbiology laboratories and the Finnish Institute for Health and Welfare (THL) [22]. THL also collects data on infections caused by notifiable bacteria (Finnish National Infectious Diseases Register) [23]. However, there is no information on the difference in resistance between domestic and travel‐associated enteropathogenic bacterial strains.

The purpose of this study was to monitor the epidemiology and antimicrobial resistance of enteropathogenic bacteria in the Hospital district of Southwest Finland over a five‐year study period from 2018 to 2022. During the study period a worldwide COVID‐19 pandemic, caused by severe acute respiratory syndrome coronavirus 2 (SARS‐CoV‐2), commenced. It has been speculated whether the risk of global dissemination of AMR bacteria and genes from endemic regions may have decreased due to reduced international travel during the pandemic [24]. Therefore, the main subject of this study was to explore whether these travel restrictions had any effect on the prevalence of domestic and travel‐associated enteropathogenic strains and AMR.

## 2 Materials and Methods

### 2.1 Strain Collection

From August 2018 to August 2022, totally 321 enteropathogenic bacterial strains were isolated from clinical fecal samples in the Clinical microbiology laboratory of Turku University Hospital, Hospital district of Southwest Finland from patients with ongoing symptoms of gastroenteritis. The fecal samples were analyzed with the BD MAX Extended Enteric Bacterial Panel (BD Life Sciences, Sparks, MD, USA), a multiplex‐PCR assay that allows identification of *Campylobacter jejuni/coli*, *Yersinia enterocolitica* , *Salmonella* sp., ETEC, *Shigella* sp./EIEC, *Vibrio* sp., Shiga toxins (STEC and *S. dysenteriae* ), and *Plesiomonas shigelloides* from the clinical fecal sample [25]. Samples positive for any of the detected enteropathogens, except ETEC, were cultured on species‐specific selective media and incubated accordingly [26]. The bacterial colonies from the culture medium were identified using the Maldi Biotyper (Bruker, Berlin, Germany) and serotyped with agglutination sera. The pure cultures of bacterial strains were stored at −80°C and were further analyzed in the Infectious Diseases and Immunity Unit, Institute of Biomedicine, University of Turku, Turku, Finland.

Samples were collected in January, February (representing long‐distance traveling during the Christmas holiday season) and August (representing the summer holiday season) over a five‐year period. Samples collected in January and February 2019–2022 are later referred to as 1–2/2019–2022, and samples collected in August 2018–2022 are later referred to as 8/2018–2022. Strains isolated from patients with a known recent travel history, that is, patients had traveled abroad within 2 weeks preceding symptoms, and this information was expressed in the referral for sampling, were considered travel‐associated and all other strains were considered domestic. Only patients who had *Salmonella* infection were specifically questioned for travel history afterwards; if not available, since the THL is serotyping only domestic *Salmonella* strains.

### 2.2 Antimicrobial Susceptibility Testing

Antimicrobial susceptibility testing (AST) was performed according to the EUCAST guidelines with disc diffusion method [27, 28]. The antimicrobial agents tested for *Campylobacter* spp. were ciprofloxacin 5 μg (CIP), tetracycline 30 μg (TET) and erythromycin 15 μg (ERY). *Campylobacter jejuni* DSM 4688 and *Campylobacter coli* DSM 4689 type strain were used as controls in AST. For *Salmonella enterica* strains, the following discs were used for AST: ampicillin 10 μg (AMP), cefotaxime 5 μg (CTX), ceftriaxone 30 μg (CRO), ceftazidime 10 μg (CAZ), meropenem 10 μg (MEM), co‐trimoxazole 1/25 μg (SXT), and pefloxacin 5 μg (PEF) was used to screen fluoroquinolone resistance. Antimicrobial agents tested for *Yersinia enterocolitica* strains were cefotaxime 5 μg (CTX), ciprofloxacin 5 μg (CIP), co‐trimoxazole 1/25 μg (SXT) and tetracycline 30 μL (TET). *Escherichia coli* ATCC 25922 type strain were used as a control strain. The antimicrobial discs were from Oxoid Ltd. (Thermo Scientific, Helsinki, Finland).

### 2.3 Detection of ESBL Production

Possible Extended‐spectrum beta‐lactamase (ESBL) ‐producing *S. enterica* strains from PCR‐positive fecal samples were screened according to EUCAST guidelines for detection of ESBLs in *Enterobacteriaceae*, that is, cefotaxime and ceftazidime disc diffusion zone being < 21 and < 22 mm, respectively [29]. DNA from possible ESBL producing strains was extracted with 5% Chelex (Bio‐Rad Finland Oy, Helsinki, Finland). Pure colonies were collected with 1 μL inoculation loop and added to 1 mL 5% Chelex. Samples were vortexed, boiled in 100°C for 30 min and centrifuged at 12,000 RPM for 12 min. Supernatant was collected and DNA was diluted to 1:10 with ultrapure PCR‐water. ESBL production was confirmed with multiplex PCR using previously published TEM, SHV and CTX‐M primers and methods [30].

### 2.4 Statistical Methods

Descriptive data was analyzed with JMP (Student Edition, v.19.0.4, JMP Statistical Discovery LLC). Binary categorical data were reported as counts and proportions in %. Group differences in numerical variables (number of strains in different time points) were tested with the Poisson exact test due to the small number of strains.

## 3 Results

**FIGURE 1:**
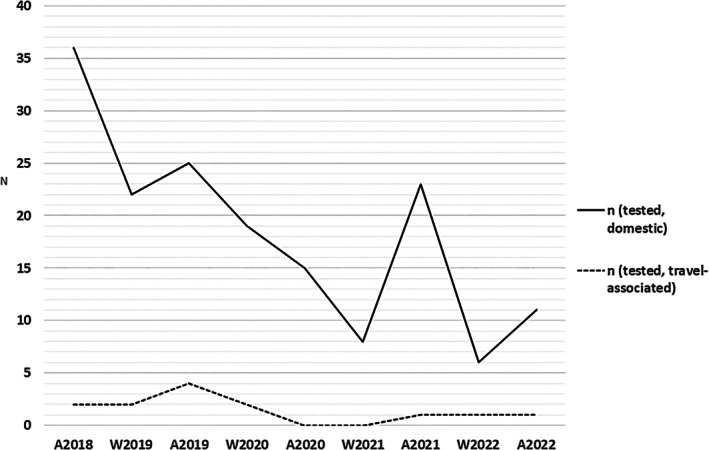
Epidemiology of domestic and travel‐associated *Campylobacter* spp. strains. A = autumn (August), W = winter (January and February).

**FIGURE 2:**
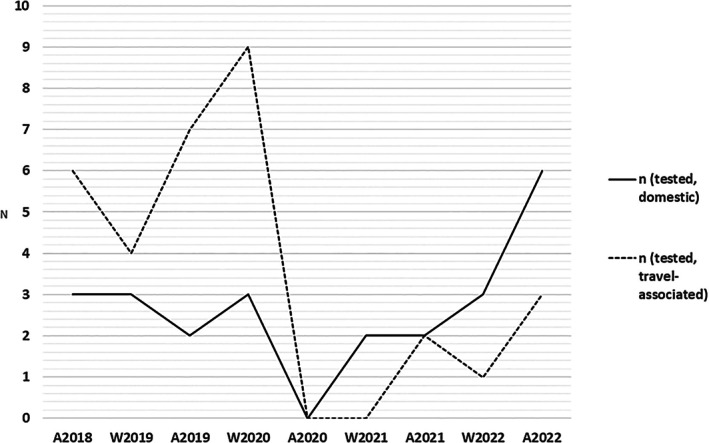
Epidemiology of domestic and travel‐associated *Salmonella enterica* strains. A = autumn (August), W = winter (January and February).

**FIGURE 3:**
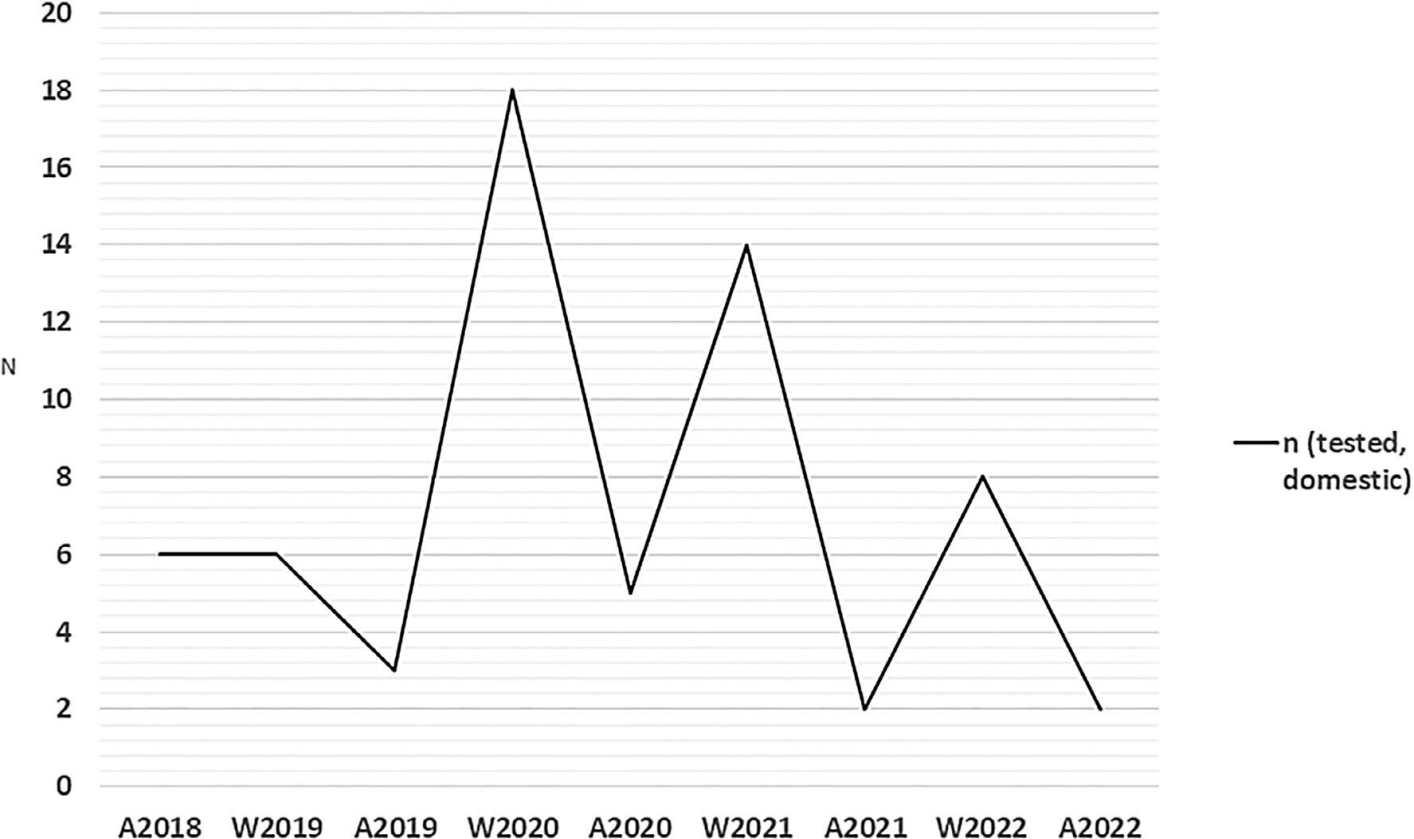
Epidemiology of domestic *Yersinia enterocolitica* strains. No travel‐associated strains were discovered. A = autumn (August), W = winter (January and February).

During the study, totally 184 *Campylobacter* (167 *C. jejuni* , 11 *C. coli* and six other *Campylobacter* spp.), 57 *Salmonella enterica* and 65 *Yersinia enterocolitica* strains were detected and isolated from PCR‐positive fecal samples. Of these 306 isolated bacterial strains, 298 (98%) were successfully recovered after storage at −80°C and overall, 178 (97%) *Campylobacter* (165 *C. jejuni* , 9 *C. coli* and four other *Campylobacter* spp.), 56 (98%) *S. enterica* and 64 (98%) *Y. enterocolitica* strains were tested for AMR. Of these strains, 85% were of domestic origin (93% of *Campylobacter* spp., 43% of *S. enterica* and 100% of *Y. enterocolitica* ) and 15% were travel‐associated. A clear decrease in travel‐associated isolates of *Campylobacter* spp. and *S. enterica* was seen between autumn 2019 and autumn 2020 (*p* = 0.0185 and *p* = 0.0018, respectively). The annual distribution of *Campylobacter* spp., *S. enterica* and *Y. enterocolitica* strains is presented in Table 1 and Figures 1, 2, 3, respectively.

**TABLE 1:** Countries of origin of *Campylobacter* spp., *Salmonella enterica* and *Yersinia enterocolitica* strains tested for antimicrobial susceptibility, and the total number of tested fecal samples at given time.

Bacterial strain	Timepoint	8/2018	1–2/2019	8/2019	1–2/2020	8/2020	1–2/2021	8/2021	1–2/2022	8/2022
*Campylobacter* spp.	Travel destination (number of strains)	Mediterranean area (1) Europe (1)	Mediterranean area (1) Southeast Asia (1)	Central Europe (3) Mediterranean area (1)	Southeast Asia (1) Mediterranean area (1)	—	—	Central Europe (1)	Unknown (1)	Mediterranean area (1)
	Travel‐associated (*n*)	2	2	4	2	0	0	1	1	1
	Domestic (*n*)	36	22	25	19	15	8	23	6	11
	Total	38	24	29	21	15	8	24	7	12
*Salmonella enterica*	Travel destination (number of strains)	Central Europe (2) Eastern Europe (1) Mediterranean area (3)	Southeast Asia (4)	Mediterranean area (4) Central Europe (2) Eastern Europe (1)	Jamaica (1) Kenya (1) Mediterranean area (1) Southeast Asia (4) Mauritius (1) Argentina (1)	—	—	Central Europe (1) Faroe Islands (1)	Norway (1)	Mediterranean area (1) Central Europe (2)
	Travel‐associated (*n*)	6	4	7	9	0	0	2	1	3
	Domestic (*n*)	3	3	2	3	0	2	2	3	6
	Total	9	7	9	12	0	2	4	4	9
*Yersinia enterocolitica*	All strains (only domestic found)	6	6	3	18	5	14	2	8	2
Total number of PCR‐tested fecal samples		531	802	449	778	358	644	431	748	374

*Note:* Mediterranean area = Spain, Cyprus, Turkey, Greece, Morocco; Southeast Asia = Thailand, Indonesia, Philippines; Eastern Europe = Russia; Central Europe = Germany, Hungary, Poland, Bulgaria, Estonia, Kosovo, Moldova, Macedonia.

AMR of *Campylobacter* spp., *S. enterica* , and *Y. enterocolitica* has varied through the study period (Tables 2, 3, 4, 5). Among *C. jejuni* , antimicrobial resistance averaged 58% and 88% to ciprofloxacin, and 36% and 63% to tetracycline, in domestic (*n* = 155) and travel‐associated (*n* = 10) strains, respectively (Table 2). Erythromycin resistance was not detected. Among *C. coli* / other *Campylobacter* spp. antimicrobial resistance averaged 90% and 67% to ciprofloxacin, 53% and 67% to tetracycline, and 67% and 0% to erythromycin, in domestic (*n* = 10) and travel‐associated (*n* = 3) strains, respectively (Table 3).

**TABLE 2:** Antimicrobial resistance profile of *Campylobacter jejuni* strains.

Antimicrobial agent		8/2018	1–2/2019	8/2019	1–2/2020	8/2020	1–2/2021	8/2021	1–2/2022	8/2022
*n* (tested)	Domestic	32	22	22	18	15	7	23	6	10
	Travel‐associated	1	1	4	2	0	0	1	0	1
CIP	R% (D)	78%	77%	68%	83%	7%	57%	17%	67%	70%
	R% (TA)	100%	100%	75%	50%	—	—	100%	—	100%
TET	R% (D)	38%	64%	23%	61%	0%	43%	4%	50%	40%
	R% (TA)	100%	0%	25%	50%	—	—	100%	—	100%

*Note:* All strains were susceptible to erythromycin.

Abbreviations: CIP, ciprofloxacin; D, domestic; R%, resistance%; TA, travel‐associated; TET, tetracycline.

**TABLE 3:** Antimicrobial resistance profile of *Campylobacter coli* /Hippurate‐negative *Campylobacter* spp.^a^ strains.

Antimicrobial agent		8/2018	1–2/2019	8/2019	1–2/2020	8/2020	1–2/2021	8/2021	1–2/2022	8/2022
*n* (tested)	Domestic	4	0	3	1	0	1	0	0	1
	Travel‐associated	1	1	0	0	0	0	0	1	0
CIP	R% (D)	50%	—	100%	100%	—	100%	—	—	100%
	R% (TA)	0%	100%	—	—	—	—	—	100%	—
ERY	R% (D)	0%	—	33%	100%	—	100%	—	—	100%
	R% (TA)	0%	0%	—	—	—	—	—	0%	—
TET	R% (D)	0%	—	67%	100%	—	100%	—	—	0%
	R% (TA)	0%	100%	—	—	—	—	—	100%	—

Abbreviations: CIP, ciprofloxacin; D, domestic; ERY; erythromycin; R%, resistance‐%; TA, travel‐associated; TET, tetracycline.

^a^ Four strains were not identified by Maldi Biotyper and thus were tested with the hippurate hydrolysis test.

Among *S. enterica* , antimicrobial resistance averaged 42% and 33% to ampicillin, 4% and 6% to cefotaxime, 0% and 4% to ceftriaxone, and 0% and 2% to co‐trimoxazole for domestic (*n* = 24) and travel‐associated (*n* = 32) strains, respectively. Of the domestic and travel‐associated strains, 33% and 45%, respectively, showed fluoroquinolone resistance. Meropenem resistance was not detected. Based on ESBL screening, three domestic and four travel‐associated strains were identified; thus, only one of the travel‐related strains was confirmed as CTX‐M positive with ESBL PCR (Table 4).

**TABLE 4:** Antimicrobial resistance profile of *Salmonella enterica* strains.

Antimicrobial agent		8/2018	1–2/2019	8/2019	1–2/2020	8/2020	1–2/2021	8/2021	1–2/2022	8/2022
*n* (tested)	Domestic	3	3	2	3	0	2	2	3	6
	Travel‐associated	6	4	7	9	0	0	2	1	3
AMP	R% (D)	33%	100%	0%	33%	—	100%	0%	67%	0%
	R% (TA)	33%	50%	29%	22%	—	—	0%	100%	0%
CTX	R% (D)	0%	33%	0%	0%	—	0%	0%	0%	0%
	R% (TA)	0%	25%	14%	0%	—	—	0%	0%	0%
CRO	R% (D)	0%	0%	0%	0%	—	0%	0%	0%	0%
	R% (TA)	0%	25%	0%	0%	—	—	0%	0%	0%
PEF	R% (D)	33%	67%	50%	33%	—	50%	0%	0%	33%
	R% (TA)	17%	25%	43%	44%	—	—	50%	100%	33%
SXT	R% (D)	0%	0%	0%	0%	—	0%	0%	0%	0%
	R% (TA)	0%	0%	14%	0%	—	—	0%	0%	0%
ESBL screening	R% (D)	0%	67%	0%	33%	—	0%	0%	0%	0%
	R% (TA)	0%	25%	29%	11%	—	—	0%	0%	0%

*Note:* AMP = ampicillin, CTX = cefotaxime, CRO = ceftriaxone, PEF = pefloxacin, used to screen fluoroquinolone resistance in *S. enterica* strains, SXT = co‐trimoxazole, ESBL screen = CTX < 21 mm and CAZ < 22 mm, R% = resistance‐%, D = domestic, TA = travel‐associated.

Among domestic *Y. enterocolitica* strains (*n* = 64) antimicrobial resistance averaged 1% to cefotaxime, 2% to ciprofloxacin, 7% to co‐trimoxazole, and 21% to tetracycline. In our study, no travel‐associated *Y. enterocolitica* were detected (Table 5).

**TABLE 5:** Antimicrobial resistance profile of domestic *Yersinia enterocolitica* strains.

Antimicrobial agent		8/2018	1–2/2019	8/2019	1–2/2020	8/2020	1–2/2021	8/2021	1–2/2022	8/2022
*n* (tested)	Domestic	6	6	3	18	5	14	2	8	2
CTX	R%	0%	0%	0%	0%	0%	0%	0%	13%	0%
CIP	R%	0%	0%	0%	0%	0%	21%	0%	0%	0%
SXT	R%	0%	0%	0%	17%	0%	0%	50%	0%	0%
TET	R %	50%	83%	0%	0%	0%	7%	50%	0%	0%

*Note:* No travel‐associated *Y. enterocolitica* strains were detected.

Abbreviations: CIP, ciprofloxacin; CTX, cefotaxime; SXT, co‐trimoxazole; TET, tetracycline.

## 4 Discussion

In the present study, we have shown that between autumn 2019 and autumn 2020 there was a clear decrease in travel‐associated strains of *Campylobacter* spp. and *Salmonella enterica* . In domestic strains, a decrease was also observed, but recovery occurred earlier especially in *S. enterica* strains. Interestingly, the COVID‐19 pandemic appears to have reduced the number of both travel‐associated and domestic *Campylobacter* spp. and *S enterica* strains, suggesting that some strains classified as domestic may, in fact, be travel‐associated. Similar phenomena have been observed across Europe: the number of *Campylobacter* and *Salmonella* infections increased in 2021 compared with 2020 but remained lower than in pre‐COVID era [6, 7]. In *Y. enterocolitica* there were no travel‐associated strains and similar decrease as in *Campylobacter* spp. and *S. enterica* was not seen in domestic *Y. enterocolitica* strains. During the study period, an overall decrease in clinical isolates of *Campylobacter* spp., *S. enterica* and *Y. enterocolitica* was observed in Southwest Finland. The same declining trend was observed in the number of PCR screened fecal samples, especially from autumn 2019 to winter 2021 (Table 1).

In *Campylobacter* spp., there was a clear decrease in travel‐associated strains from autumn 2019 to autumn 2020. The declining trend continued until winter 2021, when only eight *Campylobacter* strains were isolated, resulting in a total decrease of 79% from autumn 2018 to winter 2021 (38 to eight, *p* < 0.0001). Although *Campylobacter* infections in humans are mostly associated with poultry products, there is a known seasonal variation of *Campylobacter* infections in Finland and other Nordic countries, with domestic cases peaking in July and August [31]. This is consistent with our findings. One contributing factor to seasonality is exposure to *Campylobacter* through swimming in natural surface waters and the consumption of well water, which may become contaminated with avian fecal material [32].

In Finland, *Salmonella* infections are usually travel‐associated [17, 19], which was also seen in our study: when the travel restrictions and lockdown measures were at their strictest, the number of both travel‐associated and domestic isolates declined to zero. Travel‐associated isolates remained at zero until autumn 2021, whereas domestic isolates were found already in winter 2021. Since the number of true domestic‐origin strains in Finland is very low [18], the route of infection for domestic isolates is probably linked to imported food products [5].

During the pandemic, unlike the other enteric pathogens, the number of domestic *Y. enterocolitica* strains did not decrease clearly. However, a seasonal variation was detected also in *Y. enterocolitica* strains. The incidence of *Y. enterocolitica* strains appears to behave in the opposite manner compared with the other food‐ and waterborne pathogens in this study: the incidence is higher during the winter. The possible explanation for this is the Christmas holiday season, when lots of pork, root vegetables, and convenience foods are commonly consumed in Finland.

Travel‐associated enteric bacterial strains were not detected in our material between autumn 2020 and winter 2021. The most probable explanation for this is the restrictions on international travel due to the COVID‐19 pandemic. Additionally, the overall decreasing trend in enteric infections in Finland during the COVID‐19 pandemic can be associated with lifestyle changes that occurred during the pandemic: domestic tourism became more common, people were socially isolated, and hand hygiene was improved. A recent study has shown similar decreasing trends in the epidemiology and antimicrobial resistance in ESBL‐producing *E. coli* isolates in Finland during the pandemic [33]. It is yet to be seen if the prevalence of these bacteria will return to the same level as before COVID‐19.

The antimicrobial susceptibility of the clinically most important bacterial pathogens is monitored in Finland by the Finnish Group for Antimicrobial Resistance (FiRe), composed of clinical microbiology laboratories in Finland and the bacteriological units of THL. FiRe publishes annually a FINRES‐report on antimicrobial resistance in clinically relevant pathogens in Finland [34]. Also, the Finnish Food Authority publishes annually a FINRES‐Vet report on Finnish Veterinary Antimicrobial Resistance Monitoring and Consumption of Antimicrobial Agents [9]. The AMR status of *Campylobacter* in Finnish poultry has been fluctuating, but a positive note is that resistant *Campylobacter* strains have declined from 25% in 2018 to 3% in 2021. Due to good animal health and efficient biosecurity measures, the prevalence and AMR of *Salmonella* isolates have remained low in food‐producing animals as well as in meat and eggs [9]. Only a few reports on *Y. enterocolitica* have been published in Finland; the latest from August 2024 focused on strains isolated from small mammals and thus do not reflect the situation in clinical isolates [35]. The FiRe group does not collect data on clinical *Y. enterocolitica* epidemiology or AMR.

Based on our results, AMR in *C. jejuni* is, on average, higher in travel‐associated strains, whereas *S. enterica* shows greater year‐to‐year variation. According to the 2023 FINRES‐report [34], the resistance of fecal enteropathogenic *C. jejuni* strains to ciprofloxacin was 70% in 2019, 41% in 2020, 38% in 2021 and 56% in 2022. Our study demonstrates the same declining trend until 2021. In *C. coli* , resistance to ciprofloxacin has remained higher than in *C. jejuni* strains: in Finland, ciprofloxacin resistance in clinical fecal samples was 88% in 2019, 82% in 2020, 77% in 2021 and 82% in 2022 [34]. Our results are in line with these findings. Our data demonstrate that ciprofloxacin resistance appears to be rising again among domestic *C. jejuni* strains isolated in 2022: 67% (*n* = 4/6) in winter 2022 and 70% (*n* = 7/10) in autumn 2022 were ciprofloxacin‐resistant. Only one travel‐associated *Campylobacter* strain was found both in winter and autumn 2022, thus they both were ciprofloxacin‐resistant. Notably, among *Campylobacter* spp., no erythromycin resistance was detected in travel‐associated strains. In addition, only three strains ( *C. coli* ) were found to be multi‐resistant, and the majority of the strains were susceptible or only ciprofloxacin‐resistant. This finding suggests that antimicrobial resistance among *Campylobacter* isolates in Finland remains well controlled. However, the emergence of ciprofloxacin‐resistant *C. jejuni* strains has already jeopardized the use of fluoroquinolones as first‐line treatment of campylobacteriosis in many European countries and this should be noted also in Finland [36].

According to the 2023 FINRES report, ciprofloxacin resistance among fecal enteropathogenic *S. enterica* strains was 24% in 2019, 19% in 2020, and 14% in 2021 and 2022 [34]. In our study, pefloxacin screening found higher fluoroquinolone resistance rates among domestic (33%) and travel‐associated (45%) strains. However, in Finland, clinical laboratories screen fluoroquinolone resistance with pefloxacin disc, and ciprofloxacin resistance is confirmed with ciprofloxacin Etest, which was not performed in our study. According to the 2023 FINRES report, ceftriaxone resistance among *S. enterica* strains was 2% in 2019, 3% in 2020, and 1% in 2021 and 2022. ESBL percentage was lowest in 2021 when only 0.6% of the tested *Salmonella* strains were ESBL producers; this is in line with our results, where only one strain (1.7%) was confirmed to be an ESBL producer [34]. Our findings are consistent with these results; however, given the small sample size, AMR trends rather than exact percentages are more informative in the context of our study. Among *S. enterica* , resistance to fluoroquinolones seems to persist at a certain level, but third‐generation cephalosporin resistance seems to have reduced after the winter of 2020. Reports from European countries indicate that resistance to ampicillin and co‐trimoxazole in *S. enterica* is common, whereas co‐resistance to ciprofloxacin and cefotaxime remains uncommon [36]. By contrast, a study from Southeast Asia shows substantially higher rates of fluoroquinolone resistance and notable co‐resistance to third‐generation cephalosporins [37], raising concern that exposure via imported food products and international travel may contribute to the dissemination of antimicrobial‐resistant *S. enterica* .

### 4.1 Study Limitations

This was a sample‐based study rather than a patient‐based study; therefore, the origin of each bacterial strain was inferred from travel history information recorded in the patient information system, and no dietary or travel questionnaires were used. Consequently, the study design may underestimate the number of travel‐related strains. The study materials include bacterial strains collected from a single hospital laboratory in Finland. The Clinical Microbiology Laboratory of Turku University Hospital analyzes approximately 10% of all the clinical microbiology samples in Finland, so this represents only a subset of the national sample pool. Due to a low number of travel‐associated strains, the statistical power is limited and the results should be interpreted with caution. However, a key strength of the study is the repeated longitudinal monitoring. Finally, this study was an epidemiological study and no molecular characterization was done to study antimicrobial resistance mechanisms, thus no conclusions on transmission are made.

## 5 Conclusion

This study shows that the number of travel‐associated gastrointestinal infections caused by *Campylobacter* spp. and *S. enterica* decreased markedly from autumn 2019 to autumn 2020. The most plausible explanation is the substantial reduction in international travel caused by COVID‐19 lockdown measures. Our findings reaffirm that travel continues to be the predominant source of *S. enterica* infections in Finland. In addition, pandemic‐related restrictions appear to have curbed the introduction and spread of antimicrobial‐resistant strains in Finland.

## Ethics Statement

This study was approved by the Hospital District of Southwest Finland, research approval number T12/018/20. The Institutional Review Board of the Hospital District of Southwest Finland determined that the use of information and biospecimens recorded in a way that prevents identification of subjects is exempt from ethical committee review.

## Conflicts of Interest

The authors declare no conflicts of interest.

## Data Availability

The data supporting findings of this study are available from the corresponding author upon reasonable request.
